# Tabletability Flip in Dry Granulated Systems

**DOI:** 10.1007/s11095-025-04005-z

**Published:** 2025-12-18

**Authors:** Zijian Wang, Chenguang Wang, Tianxiang Gao, Changquan Calvin Sun

**Affiliations:** 1 Department of Pharmaceutics, College of Pharmacy, University of Minnesota, Minneapolis, MN 55455 USA; 2 Present Address: Pharmaceutical Materials Science and Engineering Laboratory, Department of Industrial and Molecular Pharmaceutics, Purdue University, Robert E. Heine Pharmacy Building, 124 C, 575 Stadium Mall Drive, West Lafayette, IN 47907 USA

**Keywords:** Bonding area, Bonding strength, Granulation, Plasticity, Tabletability flip

## Abstract

**Purposes:**

The tabletability flip phenomenon (TFP), where an active pharmaceutical ingredient (API) with poorer tabletability exhibits better tabletability when formulated with excipients, has been well documented in direct compression systems. However, the impact of granulation on TFP remains unexplored. Hence, the purpose of this work was to investigate the occurrence and underlying mechanisms of TFP in dry-granulated formulations.

**Methods:**

Acetaminophen (APAP) and ibuprofen (IBU) were used as model APIs since they exhibit TFP in non-granulated blends. Granules of each API were prepared at two porosity levels (9% and 19%) by controlling compaction pressure. Granules with and without varying levels of extragranular magnesium stearate (MgSt) were evaluated for tabletability, bonding area (BA), and bonding strength (BS).

**Results:**

For the more porous granules (19% porosity), extensive fragmentation during compaction preserved TFP through the same mechanism observed in the pre-blends. In contrast, the less porous granules (9% porosity) remained largely unfragmented during compaction, allowing their intrinsic mechanical properties to govern the BA–BS interplay. Although APAP granules showed smaller BA due to lower deformability, the higher BS led to superior tabletability, thus maintaining TFP. The incorporation of ≥ 1% MgSt minimized BS difference between formulations, effectively eliminating TFP, since the softer IBU granules exhibited higher tabletability due to larger BA.

**Conclusion:**

These results demonstrated the applicability of the BA–BS framework in explaining TFP in granulated systems and highlight the importance of controlling granule porosity and MgSt levels to optimize tabletability in dry granulation processes.

## Introduction

Although the need for non-oral drug delivery routes has increased in recent decades, driven by the rise of biologics, or large-molecule drugs, tablet dosage forms remain the most preferred option in the pharmaceutical market. Their popularity stems from ease of administration, excellent physical and chemical stability, versatile drug release profiles, high manufacturing efficiency, and low production cost [1]. Among various manufacturing methods, direct compression is often favored in tablet development because it requires only blending and compression, making it both simple and cost-effective [2]. However, successful direct compression demands adequate powder flowability and content uniformity, which can be difficult to achieve at very low or very high drug loadings [3, 4]. When these challenges arise, agglomeration or granulation techniques are routinely employed to improve flowability and ensure uniform distribution [5].

The tabletability flip phenomenon (TFP), wherein an API with worse tabletability exhibits superior tabletability upon formulation, has recently attracted increasing attention for its implications in active pharmaceutical ingredient (API) solid form selection and tablet product development [6, 7]. For example, poorly compressible solid forms should not be automatically excluded during early candidate screening, as their formulations may ultimately display enhanced tabletability. TFP is influenced by multiple variables, including excipient type, drug loading, particle properties, and tableting process parameters [8]. A predictive model based on the Vreeman–Sun equation combined with a power-law mixing rule can be used to estimate the tabletability of simple mixtures from that of pure components [9], thereby enabling the early identification of TFP during tablet formulation development [10].

**Fig. 1:**
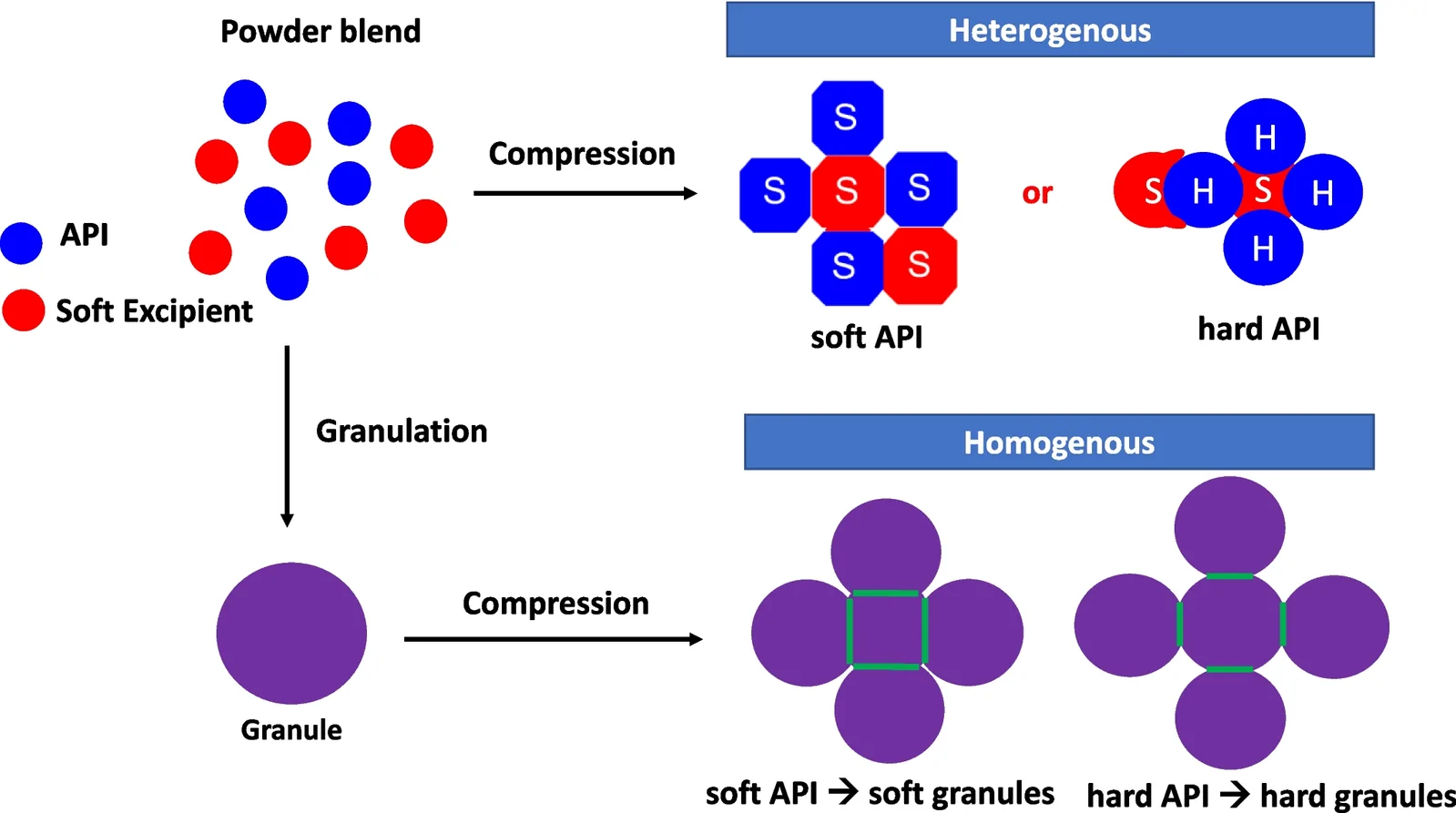
An illustration of the effect of granulation process on BA–BS interplay

The mechanisms underlying the TFP originate from the interplay between interparticulate bonding area (BA) and bonding strength (BS) [11]. BA is influenced by mechanical properties [12, 13], particle size and shape [14–17], and processing parameters [18, 19], while BS is primarily governed by the chemical nature of the components at the bonding surfaces [20]. Typically, between two APIs, the softer one exhibits better tabletability because its greater extent of plastic deformation under compression produces a larger BA [21]. However, when APIs are formulated with a soft excipient, the contact geometry between API and excipient particles becomes more complex, depending on relative plasticity. If the excipient and the softer API have comparable plasticity, a "flat" contact is formed between them, which produces a smaller BA compared to the "wrapping" contact that forms between soft excipient particles and hard API particles (Fig. 1). In this scenario, TFP arises through a BA-dominating mechanism [22]. Conversely, when the excipient is significantly softer than both APIs, it deforms sufficiently to conform to the shape of both, resulting in similar BA across the formulations. Under these conditions, TFP occurs only if the harder API provides superior BS, representing a BS–dominating mechanism [6].

To date, investigations of the TFP have focused exclusively on direct compression systems. However, it is important to explore whether TFP also occurs in granulated formulations. Granulation transforms a “heterogeneous” mixture of particles into a more “homogeneous” granule system, so the compaction behavior of granules is expected to be governed primarily by the BA–BS interplay between granules, rather than by the contact modes between individual API and excipient particles, provided extensive granule fragmentation is avoided (Fig. 1). We hypothesize that, when granules of different API formulations are prepared under the same conditions and exhibit similar porosity, granules containing the softer API will plastically deform more extensively, producing larger BA. If their BS is comparable, granules with the softer API will exhibit superior tabletability due to larger BA, effectively eliminating the TFP observed in simple powder mixtures.

In this study, we systematically investigated the impact of the dry granulation process on TFP using representative formulations containing either ibuprofen or acetaminophen. Building on the BA–BS interplay framework established for direct compression systems, we examined how granule porosity and mechanical properties influence tabletability. Specifically, we evaluated whether granules containing APIs with different plasticity, while maintained at the same porosity, exhibit differences in BA and BS that could modulate or eliminate TFP. This approach allowed us to assess whether the mechanisms identified in simple powder blends persist in granulated systems, thereby informing the selection of an appropriate manufacturing process.

## Materials and Methods

### Materials

Ibuprofen (IBU; BASF, Ludwigshafen, Germany), acetaminophen (APAP; RIA International LLC, East Hanover, NJ), microcrystalline cellulose (MCC; Pharmacel 101, DFE Pharma, Klever Strasse, Germany), lactose monohydrate (LM; Pharmatose 200 M, DFE Pharma, Klever Strasse, Germany), Crospovidone (Polyplasdone XL-10, Ashland, Wilmington, DE), and magnesium stearate (MgSt; non-bovine, HyQual™, Mallinckrodt, St. Louis, MO) were used as received.

### Methods

#### API Particle Size

Particle size distribution (PSD) of APAP and IBU was measured using a laser diffraction analyzer ((λ = 780 nm, BLUEWAVE, Microtrac, York, PA). The instrument employs a detector positioned at a fixed distance from the sample-laser interaction zone to capture scattered light over an angular range of 0.02° to 45°. Measurements were performed using the instrument setting for irregular solid particles with a refractive index of 1.51. The dispersing medium used was Isopar-G (refractive index 1.42) for APAP, and water (refractive index 1.33) for IBU. Particle size results are reported as volume-based distributions, representing the fraction of total particle volume of each size class.

#### Blend Preparation

Blends (100 g batch size) containing 20% API, 50% MCC, 24.5% LM, and 5% crospovidone were prepared using a shaker mixer (Turbula T2F, Glen Mills Inc., Clifton, NJ) at 49 rpm for 10 min, followed by an additional 1 min of mixing after adding 0.5% MgSt. These blends were then processed into ribbons, which were subsequently milled to produce granules. The resulting granules were further blended with additional MgSt at levels of 0.5%, 1%, and 2% using the same mixer at 49 rpm for 1 min.

#### True Density

The true density (${\rho }_{t}$) of MCC was obtained from the literature [23]. The ${\rho }_{t}$ values of all other powders were measured using a helium pycnometer (Quantachrome Instruments, Ultrapycnometer 1000e, Byonton Beach, FL). An accurately weighed sample was placed into the sample cell, filling approximately one-half to three-fourths of the cell volume. Each run was completed either after 50 measurements or once the standard deviation of five consecutive measurements was less than 0.005%. The average of the last five measured values was recorded as true density. The true density of mixtures (${\rho }_{t,m}$) was calculated based on the weight fraction (*f*_*i*_) and true density of each component (${\rho }_{t,i})$ using Eq. (1).

1 $${\rho }_{t,m}=\frac{1}{\sum_{i=1}^{n}\frac{{f}_{i}}{{\rho }_{t,i}}}$$

#### Preparation of Ribbons and Granules

Simulated ribbons (16 mm × 9.7 mm) with a thickness of 2–3 mm were produced using rectangular tooling on a uniaxial compaction simulator (Styl'One Evolution, MedelPharm, Beynost, France), programmed to mimic the compression profile of an Alexanderwerk WP120 roller compactor. To achieve target ribbon porosities of ~ 9% and ~ 19%, the IBU formulation was compressed at roll pressures of 10.76 kN/cm (9% porosity) and 4.9 kN/cm (19% porosity), while the APAP formulation was compressed at 14.05 kN/cm (9% porosity) and 6.0 kN/cm (19% porosity). The ribbons (~ 100 g) were milled into granules using the granulator module of a roller compactor (Alexanderwerk WP120, Germany), equipped with a pre-granulator screen (2.0 mm) and a fine granulator screen (1.2 mm). To minimize potential effects of granule size on tabletability [24], only the sieved 500–710 µm fraction was used for compaction.

#### Ribbon and Granule Properties

Ribbon porosity (ε) was determined using a caliper method [25]. Ribbon weight (*m*) was measured on an analytical balance, and a digital caliper (iGaging iP54 Fastener Cal Digital Calipers, CA) with a resolution of 0.01 mm was used to measure the ribbon length (*l*), width (*w*), and thickness (*t*). Porositywas calculated using Eq. (2), with 5 ribbons measured (n = 5).

2 $$\varepsilon =\left(1-\frac{m}{lwt{\rho }_{t}}\right)*100\%$$

Ribbon tensile strength (σ) was measured by the three-point bending method using a texture analyzer (TA-XT2i, Texture Technologies Corp., Scarsdale, NY), following the procedure described previously [26]. Tensile strength was calculated using Eq. (3), with 5 ribbons tested (n = 5).

3 $$\sigma =\frac{3Fg}{2w{t}^{2}}$$

where *F* is the breaking force, *g* is the distance between supports.

Granules were manually dispersed on a Petri dish, and images were taken using a StereoZoom microscope (Leica MZ7.5, Feasterville, PA). Captured images were analyzed using Clairity (ImageProVision Inc., Princeton, NJ), an AI-aided image analysis software, to obtain granule size distributions. Particle size is reported as the circular equivalent diameter (CED).

Granule morphology was further examined by scanning electron microscopy (SEM) (Apreo 2, Thermo Fisher Scientific, OR) operated at an accelerated voltage of 10 kV under high vacuum mode. Prior to SEM imaging, samples were sputter-coated with a 50 Å platinum layer using an ion beam sputter (IBS/TM200S, VCR Group Inc., CA).

#### Tabletability

Tablets of approximately 200 mg were prepared using a compaction simulator (Styl'One Evolution, MedelPharm, Beynost, France) programmed to mimic a Korsch XL100 press. Round, flat-faced tooling (8 mm diameter) was used for compression. Powders were compressed at pressures ranging from 10 to 350 MPa with a dwell time of 103 ms (20 rpm). Tablet dimensions were measured using a digital caliper. Tablets were then broken diametrically using a texture analyzer (TA-XT2i, Texture Technologies Corp., Scarsdale, NY) at a speed of 0.01 mm/s with a 5 g trigger force. Tablet tensile strength (σ) was calculated from the maximum breaking force (*F*), tablet diameter (*d*), and thickness (*h*) using Eq. (4), following the standard procedure [27].

4 $$\sigma =\frac{2F}{\pi dh}$$

The experimental tabletability data for each powder, i.e., tensile strength (σ) as a function of compaction pressure (*P*), were fitted to the Vreeman-Sun equation (Eq. 5) using non-linear regression to obtain the parameters σ_max_, α, and β [28].

5 $$\sigma ={\sigma }_{max}{e}^{aW(-{e}^{-\frac{P}{\beta }-1)}}$$

The ${\sigma }_{max}$ value obtained from the curve fitting, was used to evaluate the apparent bonding strength between particles or granules.

#### In-die Tablet Porosity and Heckel Analysis

In-die tablet porosity, ${\varepsilon }_{t}$, was calculated from die diameter (*D*) and in-die tablet thickness (*t*) (1 µm accuracy), ${\rho }_{t}$, and tablet weight (*m*) determined after ejection, according to Eq. (6).

6 $${\varepsilon }_{t}=1-\frac{4m}{{\rho }_{t}\pi {D}^{2}t}$$

In-die mean yield pressure (${P}_{y,i}$) was determined from a linear regression of the linear portion of the Heckel plot according to Eq. (7) [29, 30], following an established procedure [31].

7 $$-\mathrm{ln}\left({\varepsilon }_{t}\right)=\frac{1}{{P}_{y,i}}P+A$$

where *A* is the *y*-intercept of the regression line.

#### Fragmentation of Granules During Compression

Granules from both formulations, prepared at two different porosities, were first blended with 2% magnesium stearate (MgSt) using a shaker mixer at 49 rpm for 10 min to aid in distinguishing the individual granules. The resulting mixtures were then compressed into tablets at compaction pressures of 100 MPa and 300 MPa using the Styl’One compaction simulator. Tablets were manually broken, and then cross-sectional images of tablets were acquired using a Phenom XL desktop scanning electron microscope (Thermo Fisher Scientific, Waltham, MA) operated at an excitation voltage of 10 kV in low vacuum mode. Samples were mounted on a copper stage and imaged without conductive coating.

## Results and Discussion

### TFP in Simple Blends

**Fig. 2:**
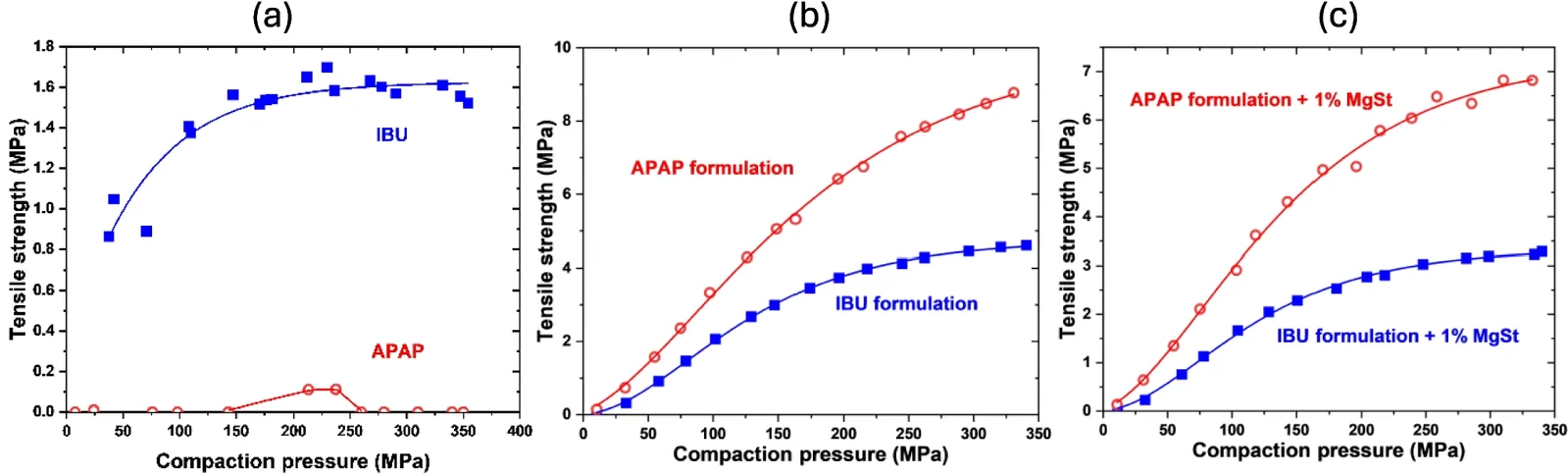
Tabletability profiles of a) pure APIs and b) API formulations, and c) API formulations containing 1% MgSt (wt%)

For the pure APIs, IBU exhibited significantly better tabletability than APAP (Fig. 2a), APAP was nearly unable to form intact tablets within the pressure range studied, whereas IBU tablets could be prepared at all pressures. The difference in tableting performance is attributed to the greater extent of plastic deformation of IBU, resulting in larger BA, as supported by the lower *P*_*y,i*_ value of IBU (28.2 ± 0.7 MPa) compared with APAP (89.5 ± 2.7 MPa) (Table 1).

**Table 1:** *P*_*y,i*_ values and tabletability parameters of samples studied in this work

Sample	*P*_*y,i*_ (MPa) (n = 3)	${\sigma }_{max}$(MPa)	*α*	*β* (MPa)
IBU	28.2 (0.7)	1.6 (0.0)	2.4 (0.7)	64.6 (19.2)
APAP	89.5 (2.7)	-	-	-
MCC	55.7 (0.5)	-	-	-
IBU formulation-powder	51.3 (1.1)	4.8 (0.0)	7.5 (0.2)	79.2 (1.9)
IBU formulation-powder + 1% MgSt	49.7 (0.3)	3.3 (0.0)	7.3 (0.4)	74.6 (3.5)
APAP formulation-powder	66.3 (0.5)	10.0 (0.2)	5.7 (0.2)	120 (5)
APAP formulation-powder + 1% MgSt	65.1 (0.4)	7.3 (0.2)	6.2 (0.5)	95.5 (7.9)
IBU -granule (19% porosity)	56.1 (0.9)	3.1 (0.1)	9.9 (2.1)	65.4 (9.3)
IBU-granule (19%) + 0.5% MgSt	55.3 (0.5)	2.7 (0.1)	9.3 (1.3)	62.6 (6.0)
IBU-granule (19%) + 1% MgSt	55.8 (0.6)	2.1 (0.0)	9.2 (0.9)	64.9 (4.1)
IBU-granule (19%) + 2% MgSt	55.1 (0.3)	1.8 (0.0)	7.4 (0.6)	61.7 (3.8)
APAP -granule (19%)	71.8 (0.4)	5.4 (0.4)	6.5 (0.7)	134 (19)
APAP-granule (19%) + 0.5% MgSt	71.2 (0.8)	3.4 (0.1)	9.0 (1.3)	72.6 (7.8)
APAP-granule (19%) + 1% MgSt	70.5 (0.4)	3.1 (0.1)	8.7 (0.7)	83.1 (5.6)
APAP-granule (19%) + 2% MgSt	70.0 (0.3)	2.4 (0.1)	6.1 (0.5)	93.9 (5.1)
IBU-granule (9%)	56.9 (0.4)	2.7 (0.1)	7.3 (0.6)	89.7 (6.7)
IBU-granule (9%) + 0.5% MgSt	55.4 (0.6)	2.0 (0.0)	7.9 (0.6)	83.4 (5.4)
IBU-granule (9%) + 1% MgSt	55.8 (0.5)	1.7 (0.0)	8.5 (0.6)	65.2 (3.3)
IBU-granule (9%) + 2% MgSt	55.2 (0.4)	1.5 (0.0)	5.7 (0.5)	93.1 (8.6)
APAP -granule (9% porosity)	69.2 (0.4)	4.6 (0.4)	6.0 (0.4)	176 (26)
APAP-granule (9%) + 0.5% MgSt	66.5 (0.2)	2.5 (0.2)	6.7 (0.9)	131 (23)
APAP-granule (9%) + 1% MgSt	65.8 (0.9)	1.9 (0.1)	7.4 (0.4)	102 (10)
APAP-granule (9%) + 2% MgSt	65.1 (0.5)	1.5 (0.1)	6.2 (0.3)	123 (8)

When both APIs were formulated with the same excipient matrix, TFP was observed as the APAP formulation, despite its initially poorer tabletability, exhibited superior tabletability than the IBU formulation (Fig. 2b). Given that the formulations contained a substantial proportion of MCC (50%, wt%) and the plasticity of MCC (*P*_*y,i*_ = 55.7 MPa) is approximately intermediate between that of IBU (*P*_*y,i*_ = 28.2 MPa) and APAP (*P*_*y,i*_ = 89.5 MPa) (Table 1), the formation of wrapping contacts is expected in both formulations. The difference is that API particles wrap MCC particles in the IBU formulation, while it is opposite in the APAP formulation. Hence, the observed TFP cannot be explained by the BA–dominating mechanism [7]. Instead, it can be attributed to the BS–dominating mechanism, as evidenced by the substantially higher (more than doubled) maximum tensile strength (${\sigma }_{max}$) achieved by the APAP formulation compared with the IBU formulation (Table 1).

Since the two APIs also differ in BS, reducing difference in BS can clarify the BA–driven TFP. MgSt is known to reduce BS, which is the primary reason for the widely reported deterioration of tabletability upon MgSt addition [32, 33]. Therefore, 1% MgSt was added to both formulations to reduce difference in BS while maintaining BA, as 1% MgSt disperses throughout the powder bed without significantly affecting particle deformation during compaction [34]. The tabletability profiles measured after MgSt addition showed that the tensile strength of both formulations decreased, confirming reduced BS (Fig. 2c). However, TFP persisted, suggesting that, under the current mixing conditions, 1% MgSt is insufficient to completely coat particle surfaces as required to eliminate difference in BS between the two systems. More extended mixing could achieve more surface coverage. Additionally, particle fragmentation during compaction may also negate the effect of MgSt coating on tabletability, even if full coverage were attained.

### Ribbon and Granule Properties

Porosity significantly influences the properties of both granules and tablets [25, 35, 36]. For a given material, ribbons with lower porosity are usually harder [36] and more difficult to mill due to a lower tendency to fragment. Consequently, granules produced from less porous ribbons are denser, harder, and larger [37]. While these granules may exhibit improved flowability, their tabletability is generally poorer because of a reduced BA [38].

To evaluate the effects of porosity on the mechanical properties of ribbons and granules, we prepared granules from ribbons with target porosities of approximately 19% or 9% for each formulation (Table 2). Target porosity was achieved by adjusting compaction pressure. Granules prepared from these ribbons are referred to as "granule (19%)" and "granule (9%)" throughout this work. As expected, ribbons with lower porosity exhibited significantly higher tensile strength within a given formulation (Table 2). At comparable porosity, APAP formulation ribbons exhibited higher tensile strength than IBU formulation ribbons, consistent with their tabletability ranking (Table 2, Fig. 2b).

**Table 2:** Ribbon properties of APAP and IBU formulations

	Compaction pressure (MPa)	Ribbon porosity (%)	Ribbon tensile strength (MPa)
APAP-ribbon	240	19.6 (0.6)	2.10 (0.10)
	560	9.0 (0.4)	7.01 (0.48)
IBU-ribbon	200	18.6 (0.5)	1.74 (0.29)
	430	9.3 (0.4)	4.75 (0.35)

**Fig. 3:**
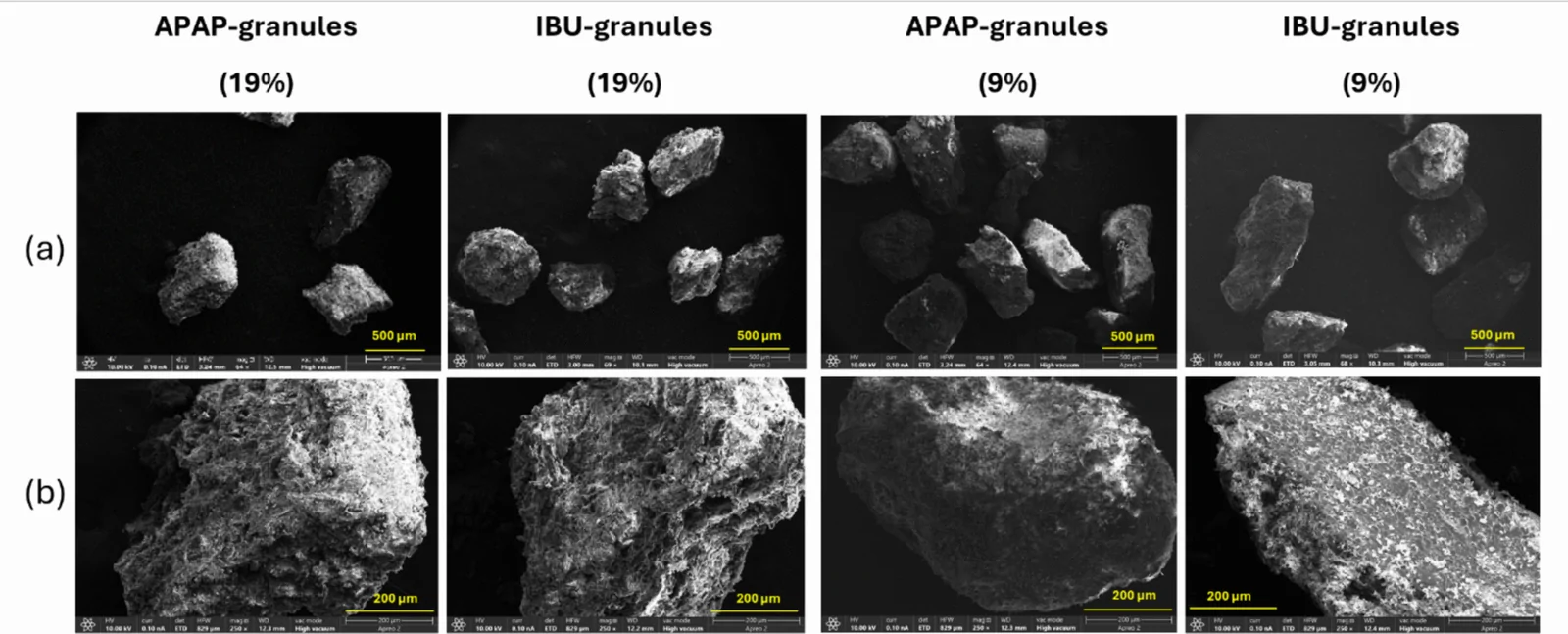
SEM images of all batches of granules at different magnifications: **a**) low (approximately × 66) and **b**) high (× 250)

To minimize potential effects of granule size on tabletability, only the 500–710 µm sieve fractions were analyzed for all granules. Within this sieve range, minimal differences in granule size distribution were observed across the four granule types (Table 3). SEM images further confirmed that the granules were similar in overall size and morphology, although granule with 9% porosity exhibited smoother and less porous surfaces than granules of 19% porosity (Fig. 3). As expected, granules are significantly larger than both APIs (Table 3).

**Table 3:** Granule size distribution of APAP and IBU containing granules

Granule	D_10_ (µm)	D_50_ (µm)	D_90_ (µm)
APAP	22.8 (1.2)	50.2 (3.9)	135.3 (7.3)
IBU	35.8 (1.6)	54.3 (3.4)	118.9 (5.2)
APAP-granule (19%)	518.6	709.0	928.3
APAP-granule (9%)	459.0	703.9	924.1
IBU-granule (19%)	530.5	705.6	909.4
IBU-granule (9%)	418.8	760.1	962.7

### TFP in High-porosity Granule (19%) System

The IBU–granules (19%) exhibited greater plasticity (*P*_*y,i*_ = 56.1 ± 0.9 MPa) than the APAP–granule (19%) (*P*_*y,i*_ = 71.8 ± 0.4 MPa) (Table 1), consistent with the intrinsically higher plasticity of IBU. Interestingly, the *P*_*y,i*_ values of granules were higher than those of the corresponding powder blends for both formulations (Table 1), suggesting that *P*_*y,i*_ reflects not only intrinsic material plasticity but is also influenced by granule structure. This topic is currently under active investigation in our laboratory.

**Fig. 4:**
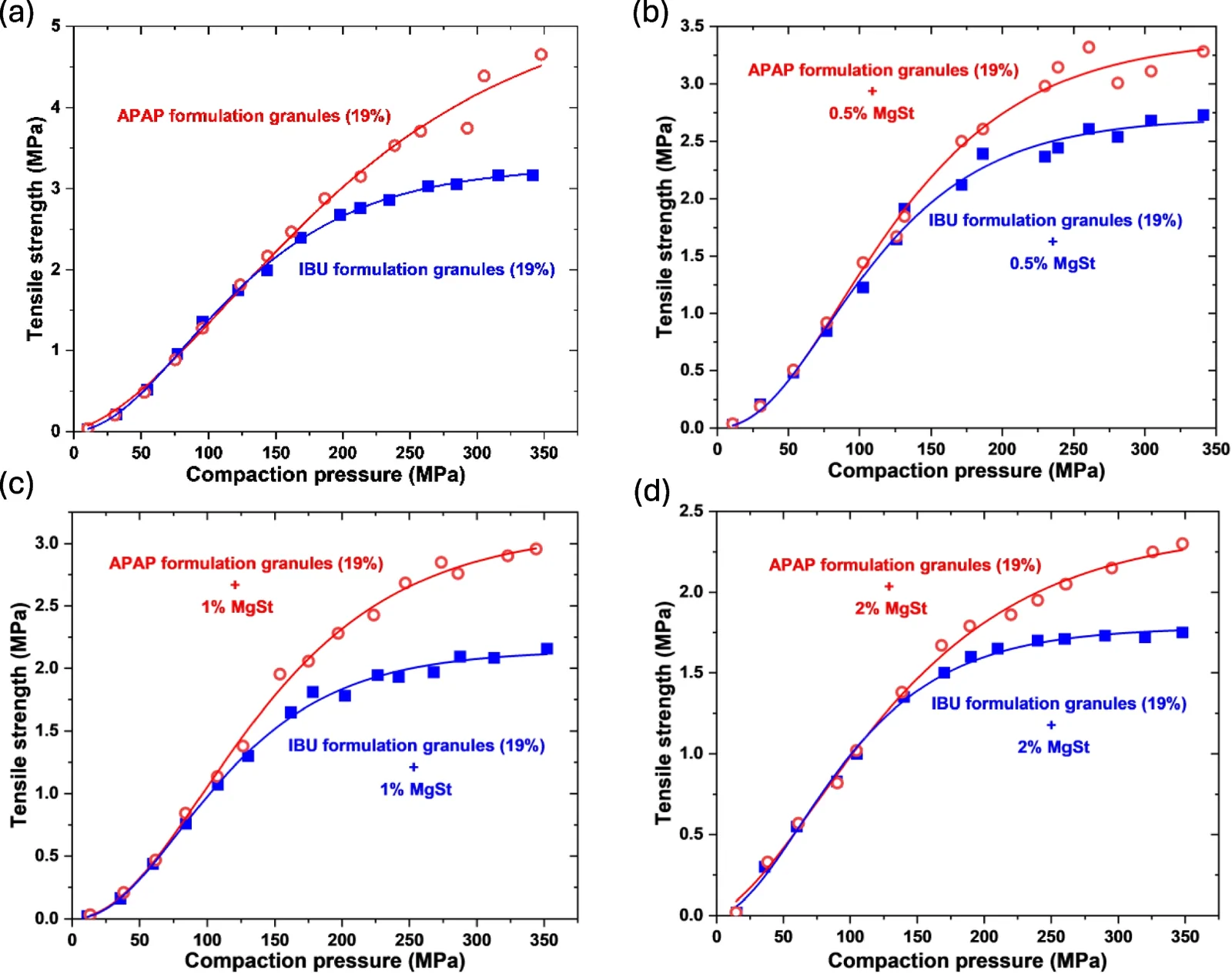
Tabletability profiles of granules (19%) a) without MgSt, and with different amount of extragranular MgSt b) 0.5%, c) 1%, and d) 2% (wt%)

Despite the higher plasticity of the IBU–granules (19%), TFP still occurred with APAP–granules (19%) exhibiting superior tabletability compared with IBU–granules (19%) (Fig. 4a), contrary to the expected trend based on plasticity. This behavior could be explained if the BS of the IBU–granules (19%) is sufficiently lower than that of APAP–granule (19%). To minimize BS difference, varying levels of extragranular MgSt were added. As expected, tabletability of both granules decreased gradually with increasing MgSt concentration, with tablet tensile strength reduced by nearly 50% at 2% MgSt (Fig. 4d). However, TFP persisted across all MgSt levels (Fig. 4b–d).

**Fig. 5:**
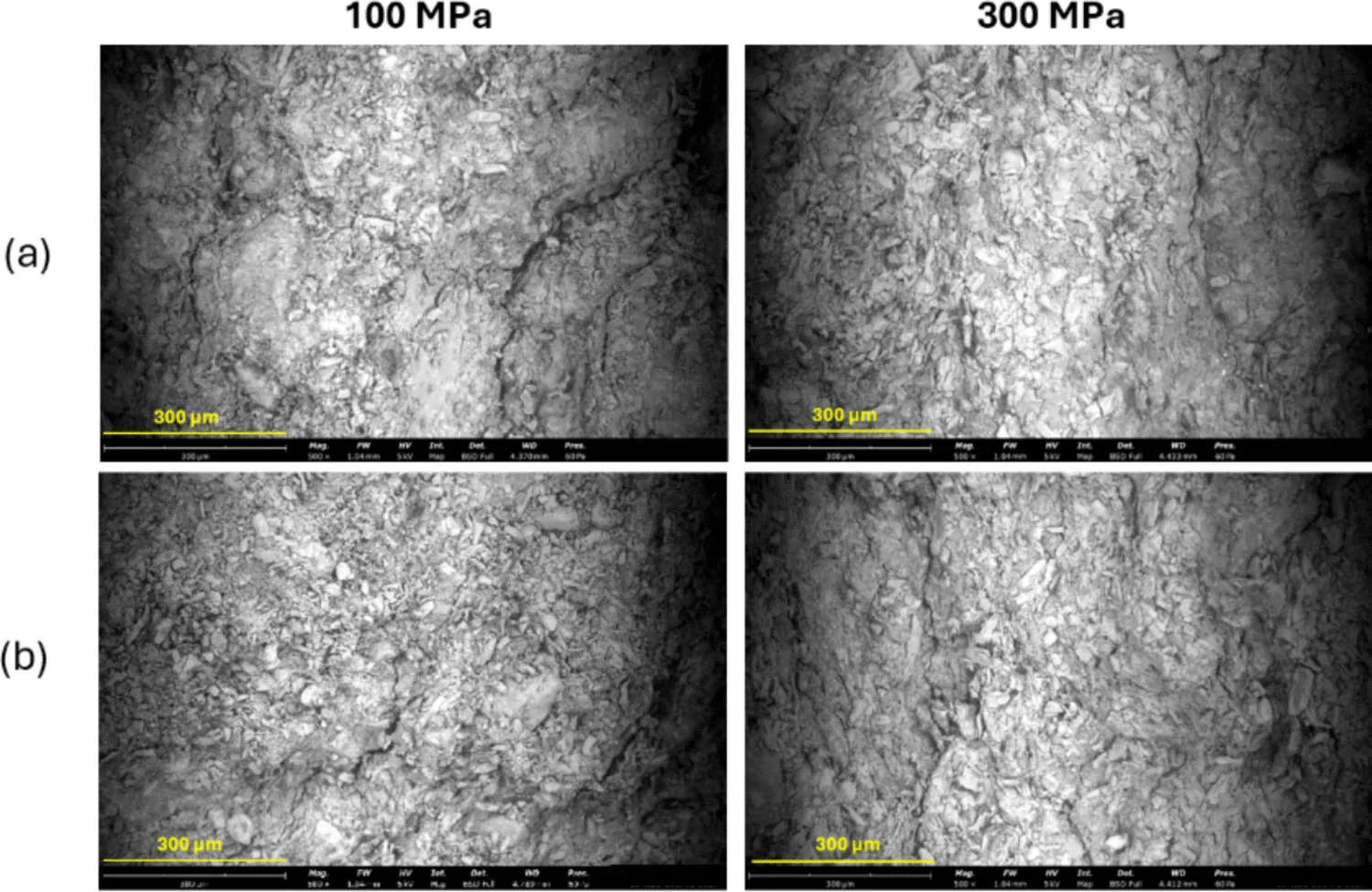
SEM images of cross-section of tablets compressed under 100 MPa and 300 MPa. a) APAP-granules (19%) and b) IBU-granules (19%). Granules were mixed with 2% MgSt (wt%) before compression

At each MgSt level (0%, 0.5%, 1%, and 2%), tabletability profiles of the two granules overlapped at low compaction pressures below a critical threshold but diverged above it (Fig. 4). This divergence can be explained by the propensity of granules to fragment during compaction. Below the critical pressure, the tabletability of IBU and APAP granules is comparable due to the interplay between BA and BS. The more plastic IBU granules undergo more extensive permanent deformation, generating larger BA. However, this BA advantage is somehow offset by the lower BS of IBU granules, leading to similar tensile strength. Above the critical pressure, granules undergo more extensive fragmentation, causing them to behave more like their original ungranulated powders, where the APAP formulation exhibits higher tabletability (Fig. 2b–c). This hypothesis is supported by SEM images of fracture surfaces of tablets obtained at compaction pressures of 100 MPa and 300 MPa (Fig. 5). Granules remain distinguished at 100 MPa but are largely unidentifiable at 300 MPa, indicating that low-pressure compaction leads primarily to granule plastic deformation, whereas high-pressure compaction induces extensive granule fragmentation.

**Fig. 6:**
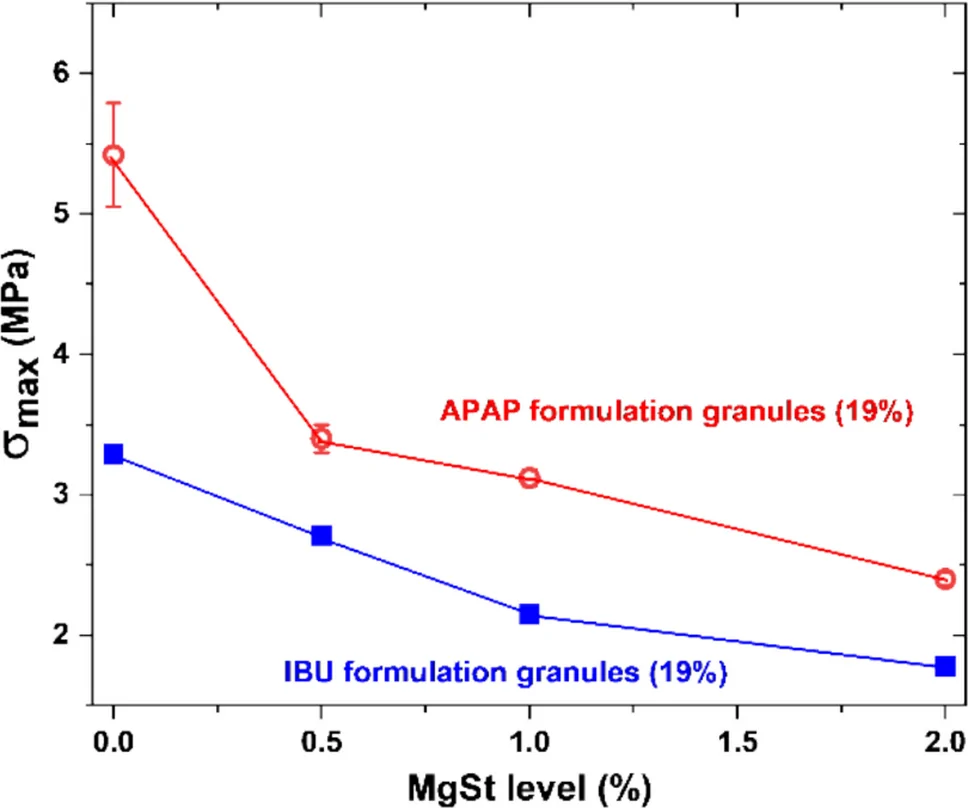
${\sigma }_{max}$ values of granules (19%) of both formulations at various extragranular MgSt levels

The apparent BS was quantified using ${\sigma }_{max}$, obtained from curve fitting the tabletability data [28]. In the absence of extragranular MgSt, the significantly higher ${\sigma }_{max}$ value of the APAP-granule (19%) than the IBU-granules (19%) indicates a much higher BS (Fig. 6). As expected, the BS of both formulations decreased upon addition of 0.5% extragranular MgSt due to the inherently low BS of MgSt. The reduction was more pronounced for the APAP formulation, narrowing the difference in BS between the two formulations. However, even with MgSt content increased up to 2%, the ${\sigma }_{max}$ values did not converge (${\sigma }_{max}$ = 2.41 ± 0.02 MPa for APAP–granule (19%) vs. 1.82 ± 0.02 MPa for IBU–granule (19%), Fig. 6), suggesting that 2% MgSt could not eliminate the inherent differences in BS between the two formulations because fracture exposed fresh surfaces for bonding.

### TFP in Low-porosity Granule (9%) System

**Fig. 7:**
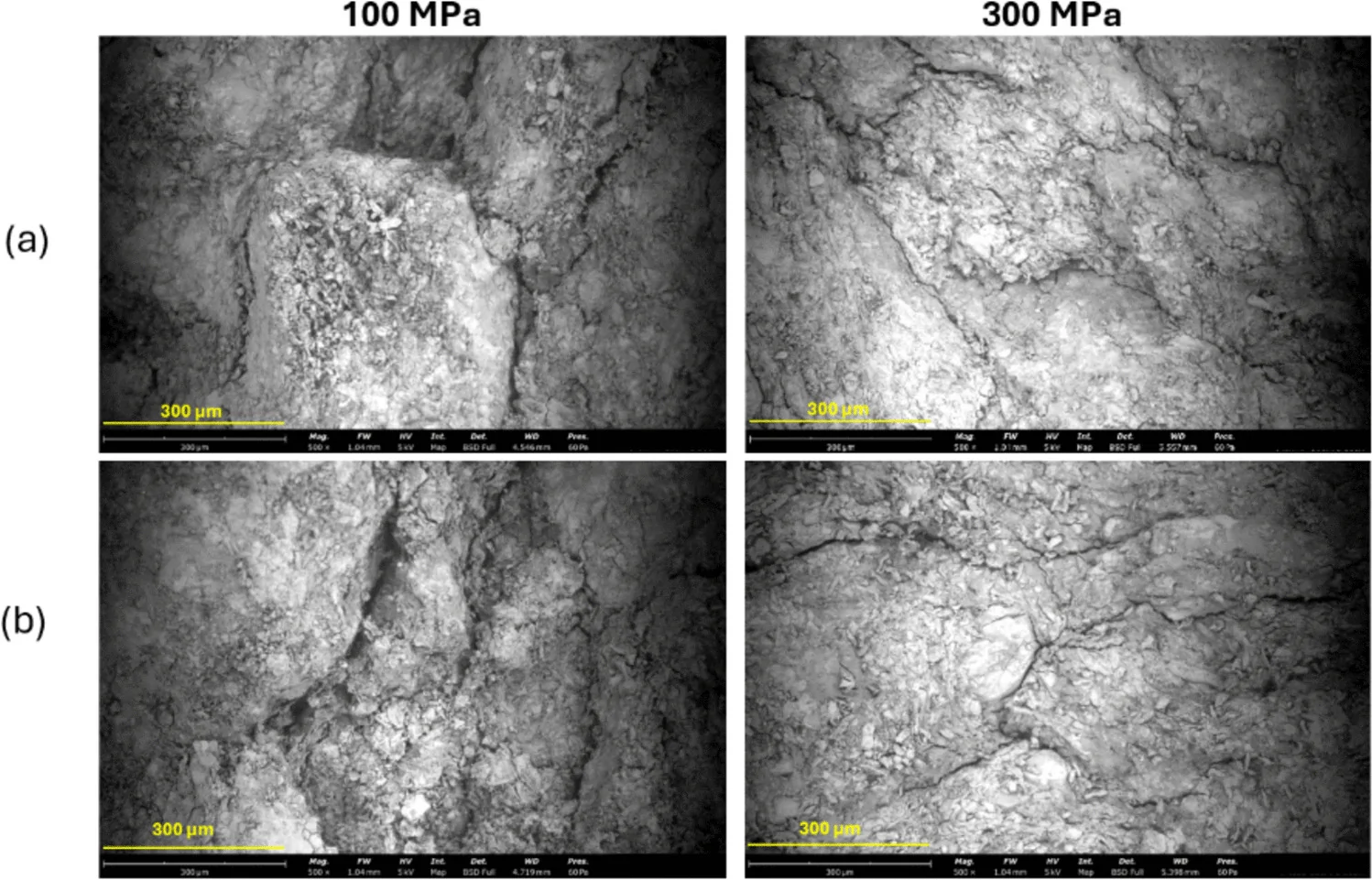
SEM images of fracture surface of tablets compressed under 100 MPa and 300 MPa a) APAP–granule (9%) and b) IBU–granule (9%). Granules were mixed with 2% extragranular MgSt (wt%) before compression

To further test the granule fragmentation hypothesis, we studied granules with lower porosity (9%) because less porous granules are more resistant to fragmentation during compaction. As expected, the IBU–granule (9%) (*P*_*y,i*_ = 56.9 ± 0.4 MPa) were more plastic than the APAP–granule (9%) (*P*_*y,i*_ = 69.2 ± 0.4 MPa). As granule boundaries were clearly visible on the fractured tablet surfaces, both APAP and IBU granules exhibited minimal fragmentation after compression at either compaction pressure (Fig. 7). Given the limited extent of granule fragmentation and the reduced intergranular BS between MgSt-lubricated granules relative to MgSt-free intragranular BS, the BA–BS interplay is predominantly governed at the granule level. At 300 MPa, the granules were more intimately bonded due to more extensive plastic deformation compared to those compressed at 100 MPa (Fig. 7), leading to larger BA and higher tensile strength.

**Fig. 8:**
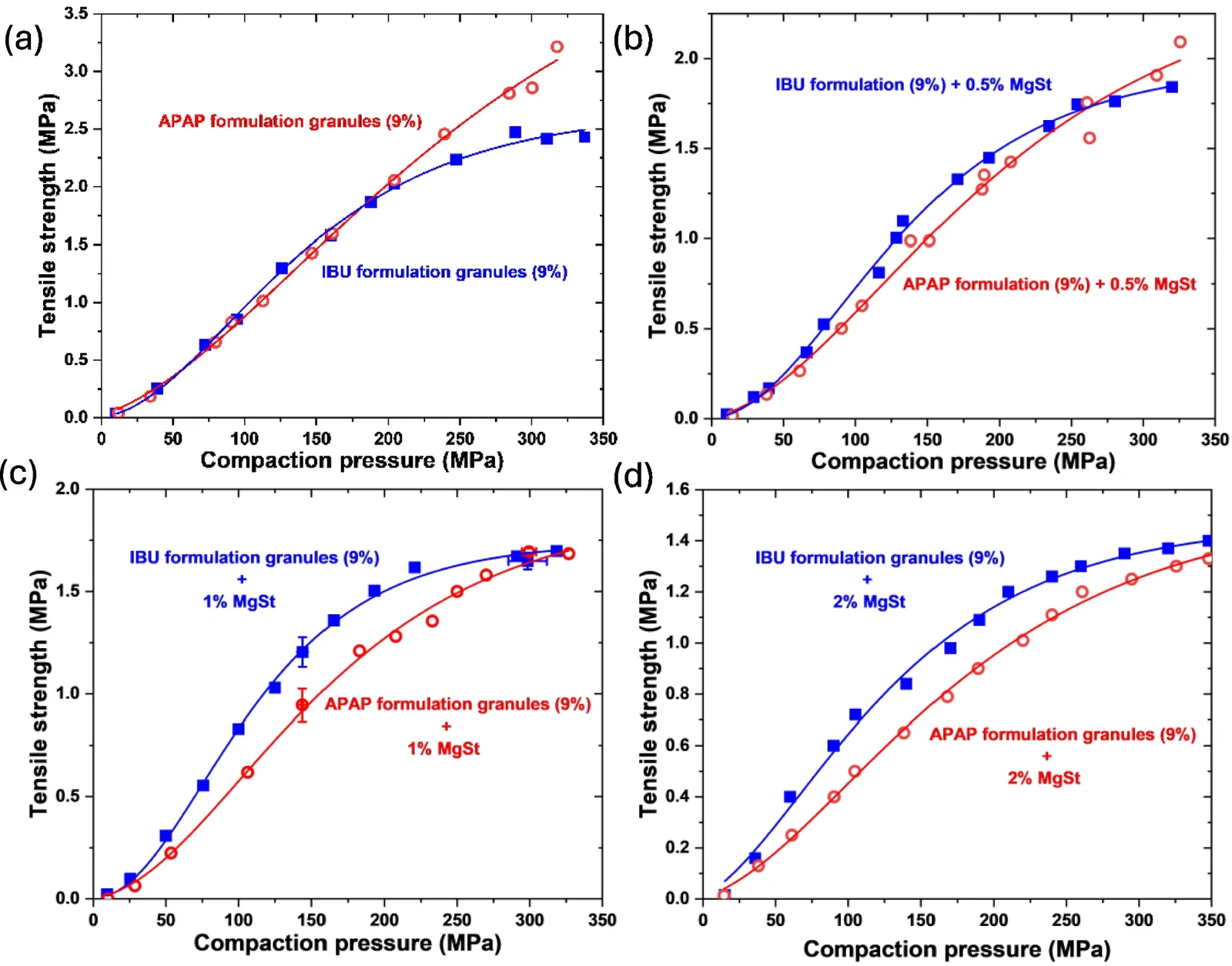
Tabletability profiles of granule (9%) from two formulations a) without MgSt, and with various amounts of extragranular MgSt b) 0.5%, c) 1%, and d) 2% (wt%)

**Fig. 9:**
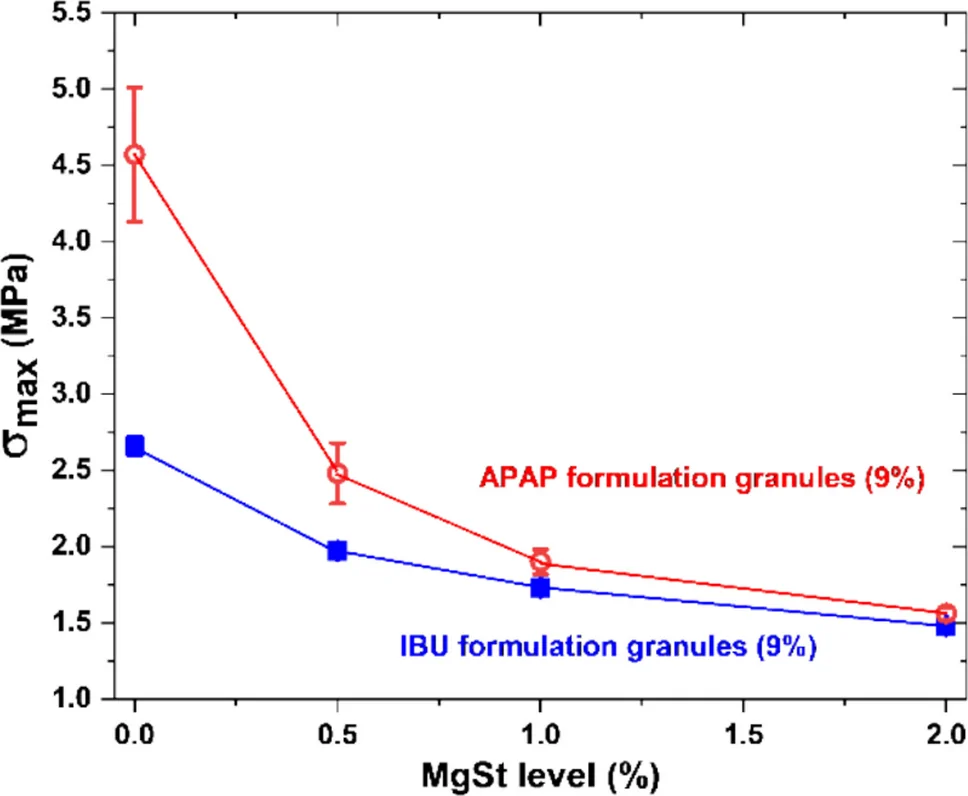
${\sigma }_{max}$ values of granules (9%) of both formulations at various MgSt levels

Although both unlubricated granules exhibited comparable tabletability below 200 MPa, the APAP-granules demonstrated superior tabletability at higher compaction pressures, and TFP persisted (Fig. 8a). This is attributed to the significantly higher BS of the APAP–granules compared with the IBU–granules (Fig. 9). Since the granules primarily remained unfragmented during compaction, the higher plasticity of the IBU–granules contributed to the formation of a larger BA than in the APAP–granules, partially offsetting the difference caused by the higher BS of APAP–granules. As a result, the tabletability difference between the two types of 9% porosity granules was smaller than that observed for the 19% porosity granules. To minimize the BS difference, varying levels of extragranular MgSt were blended with the granules. The progressive decline in tensile strength with increasing MgSt content (Fig. 8) confirms the detrimental impact of MgSt on tabletability, attributable to a reduction in BS.

At 0.5% MgSt, the tensile strength of IBU formulation tablets exceeded that of the APAP formulation below 250 MPa (Fig. 8b). However, TFP reappeared above 250 MPa, where APAP tablets exhibited higher tensile strength. This suggests that the IBU formulation granules retained lower BS and that 0.5% MgSt was insufficient to fully coat the granule surfaces, as supported by the significant difference in ${\sigma }_{max}$ value (Fig. 9). At 1% MgSt, the tabletability of the IBU formulation consistently exceeded that of the APAP formulation across the entire pressure range (Fig. 8c), indicating elimination of TFP. This outcome is attributed to the comparable BS between the two granule types (Fig. 9), while the IBU formulation granules maintained a higher BA, resulting in superior tabletability. At 2% MgSt, the IBU formulation granules continued to show better tabletability (Figs. 8d) again due to nearly identical BS (Fig. 9) and inherently higher BA.

**Fig. 10:**
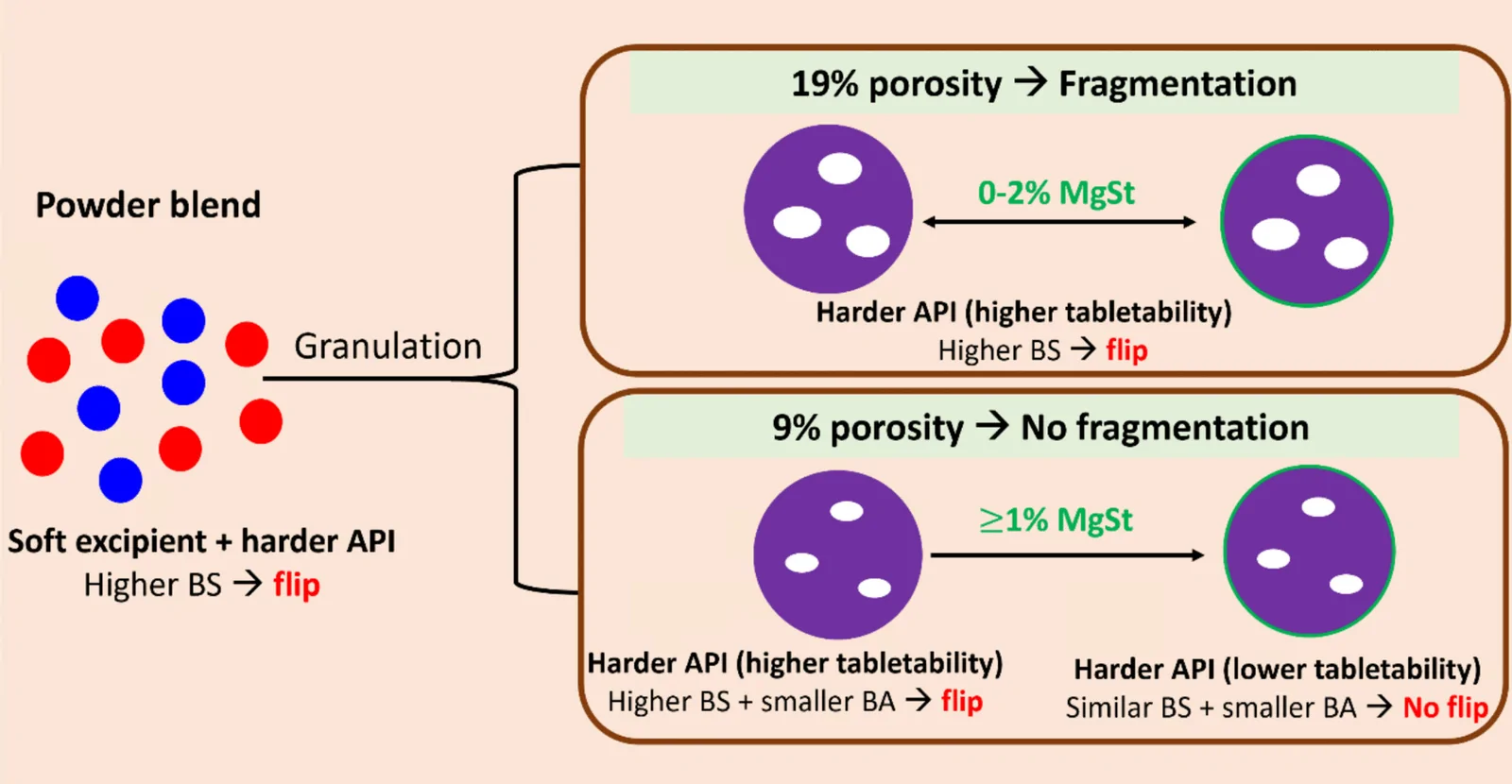
Summary of tabletability flip phenomenon in the dry granulated systems investigated in this work

The results of TFP observed in this study are summarized in Fig. 10. For the IBU–APAP system, TFP is observed in direct powder compression. After granulation, TFP can persist when granules have high porosity (19%), as extensive fragmentation during compaction transforms the initially “homogeneous” granule system into a “heterogeneous” particle system above a certain pressure threshold. In these scenarios, the harder APAP–granules exhibit higher BS and higher tabletability, resulting in TFP.

When granules have low porosity (9%) and remain largely intact during compression, they behave as a “homogeneous” system, with compaction properties governed by the BA–BS interplay at the granule level. However, TFP still persisted in this study because the harder APAP–granules had a significantly higher BS than the softer IBU–granules, despite the latter having a larger BA. The addition of MgSt was found to help normalize BS between 9%–porosity granules. When granules were blended with ≥ 1% MgSt, BS became comparable. Under these conditions, softer granules of the IBU formulation exhibited superior tabletability due to its larger BA and similar BS, effectively eliminating TFP.

## Conclusions

In this study of TFP in dry granulated systems, we found that granules containing a softer API can still exhibit lower tabletability despite their higher plasticity if extensive granule fragmentation occurs during compaction or if TFP is due to a BS-driven mechanism. TFP was eliminated when extensive granule fragmentation was minimized and when differences in granule BS between formulations were sufficiently reduced by blending with ≥ 1% MgSt. The insights from this study can guide the optimization of dry granulation processes to modulate tabletability. For example, granules of an API with poor tabletability could be designed with a higher propensity for fragmentation, such as by maintaining a higher porosity, to achieve improved tabletability.
